# The relationship between deprivation and frailty trajectories over 1 year and at the end of life: a case–control study

**DOI:** 10.1093/pubmed/fdab320

**Published:** 2021-09-20

**Authors:** Daniel Stow, Barbara Hanratty, Fiona E Matthews

**Affiliations:** Population and Health Sciences Institute, Newcastle University, Newcastle upon Tyne NE2 4AX, UK; Population and Health Sciences Institute, Newcastle University, Newcastle upon Tyne NE2 4AX, UK; Population and Health Sciences Institute, Newcastle University, Newcastle upon Tyne NE2 4AX, UK

**Keywords:** deprivation, frailty, longitudinal, socio-economic-status, trajectories

## Abstract

**Background:**

We investigated the association between area-level, multi-domain deprivation and frailty trajectories in the last year of life and over 1 year in a matched non-end-of-life sample.

**Methods:**

A 1-year longitudinal case–control study using primary care electronic health records from 20 460 people age ≥ 75. Cases (died 1 January 2015 to 1 January 2016) were 1:1 matched to controls by age, sex and practice location. Monthly interval frailty measured using a 36-item electronic frailty index (eFI: range 0–1, lower scores mean less frailty). Deprivation measured using Index of Multiple Deprivation (IMD) quintiles. We used latent growth curves to model the relationship between IMD and eFI trajectory.

**Results:**

Living in a less deprived area was associated with faster increase in eFI for cases (0.005% per month, 95%confidence interval [CI]: 0.001, 0.010), but not controls, and was associated with lower eFI at study baseline in cases (−0.29% per IMD quintile, 95%CI −0.45, −0.13) and controls (−0.35% per quintile, 95%CI −0.51, −0.20).

**Conclusions:**

Overall, greater area-level deprivation is associated with higher levels of frailty, but people who survive to ≥75 have similar 1-year frailty trajectories, regardless of area-level deprivation. Interventions to reduce frailty should target younger age groups, especially those living in the most deprived areas.

## Introduction

It is well known that people who live in deprived areas experience more ill health, worse access to health and social care services and earlier death than the rest of the population.^1–3^ There is also increasing evidence of a link between deprivation and frailty.^4–6^

Frailty is often described as a syndrome of age-related decline across multiple physiological systems, which leaves people vulnerable to a range of poor outcomes following health or other environmental stressors.^7^^,^^8^ Longitudinal studies have found differences in frailty trajectories associated with socio-economic factors such as late-life wealth,^9^^,^^10^ education,^11–13^ lifestyle and health factors^14^ and late-life income.^15^ To date, longitudinal studies exploring factors influencing frailty trajectories have measured frailty at multi-year intervals,^16^ but little is known about the impact of deprivation on changes in frailty over shorter time scales, which have been shown to be associated with an increased risk of short-term mortality.^17^

Understanding the relationship between frailty and deprivation is important to inform public health strategies to intervene in the development of frailty^18^ and to facilitate equitable access to services: particularly at the end of life, where people in lower socio-economic positions are less likely to receive high-quality care^19^ or have equitable access to hospice care^20^ than people in a higher socio-economic position.

Our primary aim was to investigate how deprivation influences short-term frailty trajectories at the end of life and if these differences could be used to inform care planning and provision at the end of life. We also sought to investigate whether any patterns we observed were unique to the last year of life or were present in non-end-of-life populations too. To address these aims we investigated the relationship between an area-level, multi-domain measure of deprivation and changes in frailty in the last year of life in people aged ≥ 75 and over 1 year in a location, age and sex-matched non-end-of-life group aged ≥ 75.

## Method

### Setting

Electronic health record (EHR) data were obtained from primary care services in UK. Data were supplied by ResearchOne (a UK based not-for-profit organization), who extract de-identified information from SystmOne (an EHR management system used by ~35% of general practices in UK and broadly representative of people registered in primary care in UK).

### Participants

People age ≥ 75 who died between 1 January 2015 and 1 January 2016 (cases). Cases were 1:1 matched to people with no record of death between 2015 and 2016 (controls). Matching criteria were date of birth (±6 months), sex and primary care practice location. Cases were excluded if their cause of death was classified as an external cause of mortality (ICD10).

### Study design

A longitudinal case–control study, with risk information collected retrospectively over 1 year. In cases, information was collected for 1-year before date of death. In controls, information was collected between January 2015 and January 2016.

### Exposure measurement

We used the Index of Multiple Deprivation (IMD) to measure area-level deprivation.^21^ IMD is the UK government’s official measure of relative deprivation in UK and uses 37 indicators across seven domains (including income, employment and education) to rank 32 844 geographical regions (‘Lowest Super Output Areas’ containing an average of 1500 people or 650 households), by relative deprivation. In our data, IMD was based on the postcode of the patient, rather than of the practice contributing data to ResearchOne. IMD was provided as the decile (rather than raw ranking), and for our analysis, we converted these to quintiles, with quintile 1 as the most deprived and quintile 5 the least deprived. If a case or a control was missing IMD information, we used the IMD value of their matched partner. Pairs where IMD was missing for the case and the control were excluded from the analysis.

### Outcome measurement

Frailty was measured using the 36-item electronic frailty index (eFI),^22^ calculated by ResearchOne at monthly intervals for 1 year from study baseline. The eFI is based on the cumulative deficit model of frailty, where an individual’s frailty score is calculated by dividing the number of deficits expressed by the individual by the total possible in the index. This creates a score between 0 (no deficits) and 1 (all deficits expressed).^23^ The eFI contains 36 deficits covering disease states (e.g. heart failure, thyroid disease), symptoms (e.g. dizziness, falls, weight loss), abnormal lab values (e.g. anaemia) and disability (e.g. visual impairment, mobility problems). The eFI is automatically generated in routine practice using an algorithm embedded in EHR management systems. The algorithm uses ~2000 Read codes (a coding system used to record patient observations in EHRs) to detect the presence of each of the 36 deficits.

### Statistical analysis

We used latent growth curve models in MPLUS^24^ to examine the relationship between deprivation quintiles and 1-year eFI trajectories. We used a model regressing age (mean-centred) and sex on the intercept only, as a baseline model (example code in Supplemental C).^25^ We measured the association between IMD quintiles and eFI trajectories by adding the effect of IMD on the intercept (baseline eFI value) and slope (rate of change in eFI over time) sequentially. In the random slopes model, intercept–slope covariance (I–S covariance) describes the relationship between baseline eFI and rate of change in eFI over time. We used sample size adjusted Bayesian information criterion to identify the best-fitting models.

We used one set of models to investigate the linear relationship between IMD quintiles as a continuous variable and baseline eFI, and another set of exploratory models using IMD quintiles as a categorical variable to test for a non-linear relationship between IMD and baseline eFI. In the categorical models, the middle/third quintile (containing the greatest number of people) was used as referent category. Separate analyses were carried out for cases and controls as the IMD is paired by the practice location matching criteria. To aid model convergence and interpretation, all eFI scores were multiplied by 100 to give a range of possible values between 0 and 100.

## Results

### Participants

Study participants were 13 149 people at end of life and 13 149 age-, sex- and practice-matched individuals not at end of life. IMD information was available for 20 460 (77.8%) participants including 11 772 (57.5%) females and 8688 (42.5%) males. Being male and younger age were associated with an increased likelihood of IMD missingness (Supplemental B). Table 1 contains demographic information for participants included in the analysis. Males were younger and had lower eFI scores than females across all IMD quintiles. The highest proportion of observations were from individuals in the middle (third) IMD quintile (*n* = 5022, 24.6%), and the most deprived (first) IMD quintile contained the fewest observations (*n* = 3117, 15.2%).

**Table 1:** Demographic information for 20 460 study participants, by case/control status and IMD quintile

			*IMD quintile*									
	*Cases*		1		2		3		4		5	
*n* (%)	**10 230**	—	**1555**	(15.20)	**1768**	(17.28)	**2514**	(24.57)	**2439**	(23.84)	**1954**	(19.10)
*n* female	**5886**	—	**911**	(15.48)	**1005**	(17.07)	**1481**	(25.16)	**1391**	(23.63)	**1098**	(18.65)
*n* male	**4344**	—	**644**	(14.83)	**763**	(17.56)	**1033**	(23.78)	**1048**	(24.13)	**856**	(19.71)
Mean age (SD)	**85.57**	(6.04)	**84.85**	(6.00)	**85.27**	(5.99)	**85.67**	(6.01)	**85.86**	(6.02)	**85.95**	(6.12)
Age female	**87.10**	(6.14)	**85.86**	(6.16)	**86.08**	(6.11)	**86.69**	(6.07)	**86.87**	(6.09)	**87.03**	(6.21)
Age male	**84.74**	(5.63)	**83.42**	(5.47)	**84.20**	(5.66)	**84.20**	(5.61)	**84.51**	(5.66)	**84.56**	(5.72)
Mean eFI (SD)	**27.71**	(11.29)	**28.20**	(11.44)	**27.86**	(11.26)	**27.87**	(11.37)	**27.48**	(11.19)	**27.29**	(11.18)
eFI female	**28.69**	(11.36)	**29.23**	(11.56)	**29.33**	(11.37)	**28.82**	(11.27)	**28.30**	(11.32)	**27.99**	(11.33)
eFI male	**26.39**	(11.05)	**26.74**	(11.13)	**25.92**	(10.82)	**26.50**	(11.37)	**26.39**	(10.94)	**26.41**	(10.93)
	**Controls**											
*n* (%)	**10 230**	—	**1562**	(15.27)	**1759**	(17.19)	**2508**	(24.52)	**2454**	(23.99)	**1947**	(19.03)
*n* female	**5886**	—	**914**	(15.53)	**1001**	(17.01)	**1469**	(24.96)	**1409**	(23.94)	**1093**	(18.57)
*n* male	**4344**	—	**648**	(14.92)	**758**	(17.45)	**1039**	(23.92)	**1045**	(24.06)	**854**	(19.66)
Mean age (SD)	**86.10**	(6.04)	**85.34**	(6.00)	**85.78**	(5.97)	**86.19**	(5.99)	**86.44**	(6.05)	**86.44**	(6.11)
Age female	**86.57**	(6.13)	**86.34**	(6.19)	**86.65**	(6.11)	**87.21**	(6.02)	**87.45**	(6.13)	**87.52**	(6.21)
Age male	**84.23**	(5.64)	**83.93**	(5.43)	**84.63**	(5.57)	**84.73**	(5.64)	**85.09**	(5.68)	**85.05**	(5.70)
Mean eFI (SD)	**23.87**	(11.11)	**24.70**	(11.50)	**23.80**	(11.06)	**23.70**	(10.98)	**23.67**	(11.04)	**23.73**	(11.08)
eFI female	**25.18**	(11.33)	**25.80**	(11.72)	**25.00**	(11.25)	**25.03**	(11.23)	**25.08**	(11.25)	**25.16**	(11.29)
eFI male	**22.09**	(10.56)	**22.21**	(11.00)	**21.82**	(10.60)	**21.76**	(10.33)	**21.90**	(10.45)	**25.80**	(10.53)

### Statistical analysis

#### Model selection

In both the end-of-life and non-end-of-life samples, model 2 (Supplemental A: random intercept and fixed slopes) produced the best fit. In the end-of-life sample, regressing a change in eFI over time on IMD did not improve model fit (Supplemental A: model 2 versus model 3) but we did observe a small effect of IMD quintile (as a continuous linear predictor) on slope (Supplemental A, model 3). Here the regression coefficient for IMD on slope meant a 0.01% per month increase in eFI for each increase in IMD quintile (95% confidence interval [CI]: 0.00, 0.01). IMD was associated with the first (baseline) measurement of eFI showing a linear trend with IMD quintile.

To facilitate comparison, we present the models that regressed IMD (categorical and linear) on the intercept term only for cases and controls (Supplemental A: model 2—results displayed in Table 2).

**Table 2:** Model parameters and regression coefficients from the best-fitting latent growth curve model in control data, with comparison to the same model (2) specified in case data

	*IMD quintile as a continuous linear predictor*				*IMD quintile as a categorical predictor*			
	*Controls*		*Cases*		*Controls*		*Cases*	
Model parameters								
	Mean	95% CI	Mean	95% CI	Mean	95% CI	Mean	95% CI
Intercept (I)	23.57	22.99, 24.15	27.69	27.08, 28.29	22.25	21.77, 22.73	27.14	26.59, 27.69
Slope (S)	0.14	0.13, 0.14	0.25	0.25, 0.26	0.14	0.13, 0.14	0.26	0.25, 0.27
I–S covariance	−0.02	−0.70, 0.02	−0.64	−0.71, −0.57	−0.02	−0.94, 0.35	−0.75	−0.83, −0.67
Regression coefficients								
Parameter	Intercept	95% CI	Intercept	95% CI	Intercept	95% CI	Intercept	95% CI
Female	1.76	1.35, 2.19	1.39	0.95, 1.83	1.77	1.35, 2.19	1.51	1.03, 1.99
Age	0.57	0.53, 0.60	0.37	0.33, 0.40	0.57	0.54, 0.60	0.37	0.33, 0.41
IMD quintile	−0.35	−0.51, −0.20	−0.29	−0.45, −0.13	—	—	—	—
IMD Q1	—	—	—	—	1.46	0.80, 2.13	0.48	−0.28, 1.24
IMD Q2	—	—	—	—	0.34	−0.30, 0.98	0.06	−0.67, 0.79
IMD Q3	—	—	—	—	ref	ref	ref	ref
IMD Q4	—	—	—	—	−0.17	−0.76, 0.41	−0.28	−0.95, 0.38
IMD Q5	—	—	—	—	−0.11	−0.73, 0.51	−0.69	−1.40, 0.02

#### Model description

In the end-of-life sample, mean baseline eFI was higher (27.7%, 95%CI: 27.1, 28.3) and increased more quickly over the year, at an average of 0.25% (95%CI: 0.25, 0.26) per month. In the non-end-of-life sample, mean eFI at baseline was 23.6% (95%CI: 22.0, 24.2) and increased by an average rate of 0.14% per month (95%CI: 0.13, 0.14) over the year.

Table 2 shows that the coefficient for the linear association between IMD and baseline eFI was larger in the non-end-of-life group than in the end-of-life group, decreasing by 0.35% (95%CI: −0.51, −0.20) per IMD quintile in the non-end-of-life sample, versus 0.29% (95%CI: −0.45, −0.13) in the end-of-life group. Significant intercept–slope covariance (I–S covariance) in the end-of-life group (−0.64 95%CI −0.71, −0.57) suggests a degree of convergence in slopes over time (meaning individuals with higher baseline scores accrue deficits more slowly than those with lower baseline scores).

**Fig. 1:**
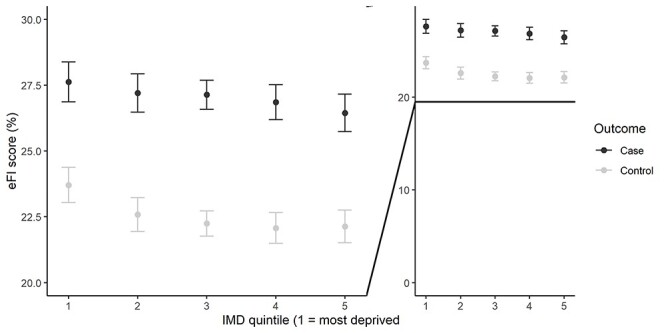
Age- and sex-adjusted eFI scores at study baseline using the results of the model regressing intercepts on IMD quintiles as a categorical variable (right provides context to the magnitude of the observed effects).

Figure 1 shows the results from models using IMD quintile as a categorical predictor of baseline eFI scores. In the non-end-of-life sample, people in the most deprived quintile (IMD Q1) had higher baseline eFI than controls in the reference quintile (IMD Q3) by 1.5% (95%CI: 0.8, 2.1). Estimates for the relationship between baseline eFI and other IMD quintiles were close to zero, with wide CIs. In the end-of-life sample, point estimates for the regression of IMD on intercepts showed a similar trend across all quintiles, but as expected with no improvement in model fit, all CIs were wide and overlapping.

## Discussion

### Main finding of this study

We found limited evidence of an association between lower area-level multi-domain deprivation and faster rate of change in eFI scores for people age 75+ in the last year of life. We did not find evidence of an association between deprivation and longitudinal frailty trajectories in our control group. In a cross-sectional analysis, we found that higher baseline levels of frailty were associated with greater area-level disadvantage in both cases and controls after adjusting for age and sex. In controls, we found some evidence of a non-linear relationship, and the largest disparity was seen in people in the most deprived quintile. For cases we observed a smaller linear trend, more evenly distributed across all quintiles.

### What is already known on this topic

Previous studies have reported that a range of factors related to deprivation are associated with differences in frailty and frailty trajectories over time, albeit often in younger cohorts than we have studied here.^4–6^^,^^9–15^

Direct comparison between our results and other studies are on frailty index trajectories are challenging due to the variety of tools used to measure frailty and markers of deprivation, as well as the statistical methods employed to model trajectories.^16^ Our findings are similar to those of Chamberlain and colleagues, who found that social and behavioural factors did not influence 8-year frailty trajectories in older (80+) age groups.^26^ Existing work using the eFI has suggested a larger association between IMD and single time point measures of eFI than we report here,^22^,^27^ but these studies included younger participants (and analyses were not age or sex adjusted).

### What this study adds

To our knowledge, this is the first study to look the relationship between area-level deprivation and 1-year frailty trajectories. We observed small effects that, clinically, are unlikely to be meaningful at the individual level. However, at the population level, our findings suggest that living in a disadvantaged area is associated with a higher accumulation of deficits over the life course, but that the effect of multi-domain, area-level deprivation on short-term trajectories of frailty in people aged ≥ 75 is limited. The slightly stronger association between IMD and baseline eFI in controls than in cases suggests that the effects of deprivation may diminish as people approach death.

The small longitudinal effect we observed in the last year of life for people near end of life may be due to greater availability of or access to end-of-life care for people living in less deprived areas. It is recognized that a range of factors (such as income, education, occupation, housing quality and area-based deprivation) reduces the likelihood that someone will access or have access to end-of-life care services that could help to improve their quality of life as they approach death.^19^

Our study used primary care data, and it is known that more affluent areas attract proportionally more general practitioners to serve their populations.^28^^,^^29^ Greater availability of services in these areas means people in less deprived areas are more likely to have contact with healthcare professionals, who recognize and code the deficits that make up the eFI. This may also have implications for healthcare record-based automated tools designed to alert clinicians to patients nearing end of life as, at a population level, automatically generated metrics (such as the eFI) might underestimate frailty in people living in deprived areas. Alerts based on changes in frailty scores would be more likely in less deprived areas and scores derived for patients who are seen more regularly would more accurately reflect the individual’s health status.

Our results may also reflect morbidity compression,^30^ as people living in the most deprived areas who survived to 75+ did so with higher levels of frailty than people living in the less deprived areas. These differences were not as pronounced in the last year of life, and along with the small effect of deprivation on trajectory we observed in people in the last year of life suggest people living in less deprived areas accrue deficits over a shorter timeframe than people living in more deprived areas.

Taken as a whole, our findings point to a need to intervene and address frailty earlier in the life course, especially where people are living in more deprived areas. Midlife in particular is emerging as an important phase for frailty development.^31^^,^^32^

### Limitations of this study

We used data from a nationally representative primary care database, but fewer people from areas of greater deprivation were present in our dataset. This is most likely to be the result of a selection bias induced by our study entry criteria (75 years). The difference in life expectancy between people living in the least and most deprived areas of UK means that fewer people from deprived areas will have survived to our study entry age (75 years).^33^

This observation may also be a result of the geographical distribution of SystmOne practices contributing to the ResearchOne database. All primary care databases in UK have some degree of regional bias (related to regional uptake and market share of the live systems from which these research databases are derived). At the time of data extraction for this study, ResearchOne was found to be more geographically representative than the CPRD and THIN databases but was likely to underrepresent some areas in the North West, West Midlands, London and South East and overrepresent areas in the East of UK and some southern regions.^34^

There were many records with missing IMD values in our dataset. These have been excluded from analysis in previous research using data from the SystmOne platform, as this indicates poor data quality.^35^ If an association between area-level disadvantage and data quality exists, this may also account for the relatively smaller number of people living in the most deprived areas in our sample. In this study, we were not able to discern whether IMD missingness was related to geographical location (or IMD group), but being male and younger increased the likelihood of IMD information being missing. This missing information may have lowered the precision of our estimates and reduced our ability to detect small effects in the most deprived groups. This is a limitation of our approach, but this reflects ‘real world’ routinely collected primary care data and as such our study shares this limitation with other studies based on primary care data.

Our findings may be sensitive to the exposure and outcome measures used. Recent work found that eFI correlated strongly with the Edmonton frail scale, but only moderately with the Clinical Frailty Scale and the frailty phenotype.^36^ Our use of IMD deciles (converted to quintiles) may mask some specific effects of the individual domains the IMD is based upon (for example, geographical areas in the same IMD quintile may be very different in terms of education or wealth). Individual socio-economic characteristics are not routinely recorded in primary care, and therefore, we were not able to control for individual-level socio-economic characteristics. We were not able to determine the impact of place on frailty or frailty trajectories, which can play an important role in mediating life expectancy,^2^ and we were unable to examine the interaction between IMD frailty and mortality risk because our methods produced closely matched case and control IMD information.

Ranks in the IMD are ordered but are not scalar (i.e. the distance between ranks may not be the same). As a result, IMD cannot be used to infer whether absolute deprivation has increased or decreased over time, meaning direct comparisons between this work and potential future studies would be limited. As the IMD rank is calculated per area, and relative to other areas, it is possible that groups of deprived individuals may live in non-deprived areas and vice versa—hence (and in common with all studies using area-level measures of deprivation), our findings are liable to the ecological fallacy.

## Conclusion

Our findings suggest there is only a small effect of area-level deprivation on the rate of change in frailty over short time scales for people age ≥ 75. However, at any given point in time, people living in more deprived areas live with higher levels of frailty, and in the general population, this effect is most pronounced for people living in the most deprived areas. In the last year of life, the association between area-level deprivation and frailty is smaller. We suggest that differences in frailty develop earlier in life and that research on, and interventions to reduce, frailty should be targeted at younger ages, particularly for people living in the most disadvantaged areas.

## Supplementary Material

2021-05-07_-_SUPPLEMENTAL_A_fdab320

2021-05-07_-_SUPPLEMENTAL_B_fdab320

2021-05-07_-_Supplemental_C_fdab320

## Data Availability

The data that support the findings of this study are available from ResearchOne, but restrictions apply to the availability of these data, which were used with appropriate permissions for the current study, and so are not publicly available. Data are not available from the authors because they relate to real and current NHS health records, and the authors do not have permission to share these data.

## Notes

**Daniel Stow**, Research Associate

**Barbara Hanratty**, Professor of Primary Care and Public Health

**Fiona E. Matthews**, Professor of Epidemiology
