# Supplementary material for: The relationship between deprivation and frailty trajectories over 1 year and at the end of life: a case–control study
Source: J Public Health (Oxf). 2021 Sep 20;44(4):844–50. doi: 10.1093/pubmed/fdab320 (PMC9715292; doi:10.1093/pubmed/fdab320)
Supplement: 2021-05-07_-_SUPPLEMENTAL_B_fdab320 [file 2021-05-07_-_supplemental_b_fdab320.docx]

5838 (22.2%) IMD values were missing in the full dataset. Missing IMD values were associated with male sex, and younger age. Supplemental figure 1a shows that a higher percentage of men were missing IMD scores than were women. Supplemental figure 1b shows that the mean age of people missing IMD scores was slightly lower than those with IMD scores

**Supplemental figure 1 a) Bar chart of missingness by sex b) Boxplot of participants' ages by missingness group**


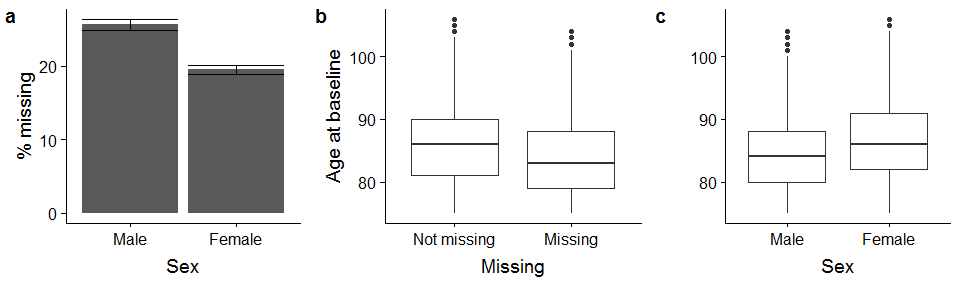


Logistic regression for IMD missingness by mean-centred age and sex (Supplemental table 1) suggested that women were 20% less likely to be missing IMD scores, and for every year of age people were less likely to be missing IMD scores by 5%.

Supplemental table 1 Results of logistic regression of IMD missingness predicted by age and sex

| Predictor | OR | 95%CI | p value |
| --- | --- | --- | --- |
| Age | 0.95 | (0.95 - 0.95) | < 0.001 |
| Female | 0.79 | (0.77 - 0.80) | < 0.001 |
| OR - Odds ratio, CI - Confidence interval | | | |
