# Supplementary material for: The relationship between deprivation and frailty trajectories over 1 year and at the end of life: a case–control study
Source: J Public Health (Oxf). 2021 Sep 20;44(4):844–50. doi: 10.1093/pubmed/fdab320 (PMC9715292; doi:10.1093/pubmed/fdab320)
Supplement: 2021-05-07_-_Supplemental_C_fdab320 [file 2021-05-07_-_supplemental_c_fdab320.docx]

**TITLE:**

Example MPLUS code for latent growth mixture model with fixed slopes and random intercepts using IMD quintile as a categorical variable

**DATA:**

File is

"Data_file.csv";

**VARIABLE:**

NAMES = id female age_c outcome decile m0 m1 m2 m3 m4 m5 m6 m7 m8 m9 m10 m11 m12; *!declaring variables in the dataset mX…. Are eFI measurements at months 0 (baseline) -12 (study end / month of death)*

IDVARIABLE IS id; *!declare the ID variable column*

USEVARIABLES ARE id female age_c m0 m1 m2 m3 m4 m5 m6

m7 m8 m9 m10 m11 m12 imd_q1 imd_q2 imd_q4 imd_q5; *!specifies variables to be included in the model*

USEOBSERVATIONS are outcome==1 AND imd_miss==0 *! outcome filters for end of life people (outcome =1), imd_miss=0 filters for people with IMD information (excludes missing)*

**DEFINE:**

*!this creates dummy variables for IMD categories with the group containing the largest number of observations (deciles 5 and 6) as the reference category*

imd_q1=0;

IF(decile == 1 OR decile ==2) THEN imd_q1=1;

imd_q2=0;

IF (decile == 3 OR decile ==4) THEN imd_q2=1;

imd_q4=0;

IF (decile == 7 OR decile ==8) THEN imd_q4=1;

imd_q5=0;

IF (decile == 9 OR decile ==10) THEN imd_q5=1;

**ANALYSIS:**

TYPE = GENERAL;

ESTIMATOR = ML;

ITERATIONS = 100000;

CONVERGENCE = 0.00005;

**MODEL:**

i s |m0@0 m1@1 m2@2 m3@3 m4@4 m5@5 m6@6 m7@7 m8@8 m9@9 m10@10 m11@11 m12@12; !defines the x values for time varying measure y (eFI)

i WITH s; *!explicitly requests intercept-slope covariance*

i ON age_c; *!regress intercept on age*

s ON age_c@0; *!fixed slope model*

i ON female; *!regress intercept on female*

s ON female@0; *!fixed slope model*

i on imd_q1; *!regress intercept on IMD quintile 1*

s on imd_q1@0; *!fixed slope model*

i on imd_q2; *!regress intercept on IMD quintile 2*

s on imd_q2@0; *!fixed slope model*

i on imd_q4; *!regress intercept on IMD quintile 4*

s on imd_q4@0; *!fixed slope model*

i on imd_q5; *!regress intercept on IMD quintile 5*

s on imd_q5@0; *!fixed slope model*

**OUTPUT:**

SAMPSTAT TECH1 TECH2 TECH3 TECH4 CINT; *!requests mplus output – CINT = confidence intervals*

**PLOT:**

TYPE IS PLOT3;

SERIES m0(0) m1(1) m2(2) m3(3) m4(4) m5(5) m6(6) m7(7) m8(8) m9(9) m10(10)

m11(11) m12(12); *!defines the plot x axis*
